# Supplementary figures and images for: Sphingolipid and ceramide associations with tau pathology vary across diverse ethnoracial groups in postmortem brain tissue
Source: medRxiv. 2025 Nov 7:2025.11.04.25339489. Preprint. [Version 3] doi: 10.1101/2025.11.04.25339489 (PMC12637755; doi:10.1101/2025.11.04.25339489)

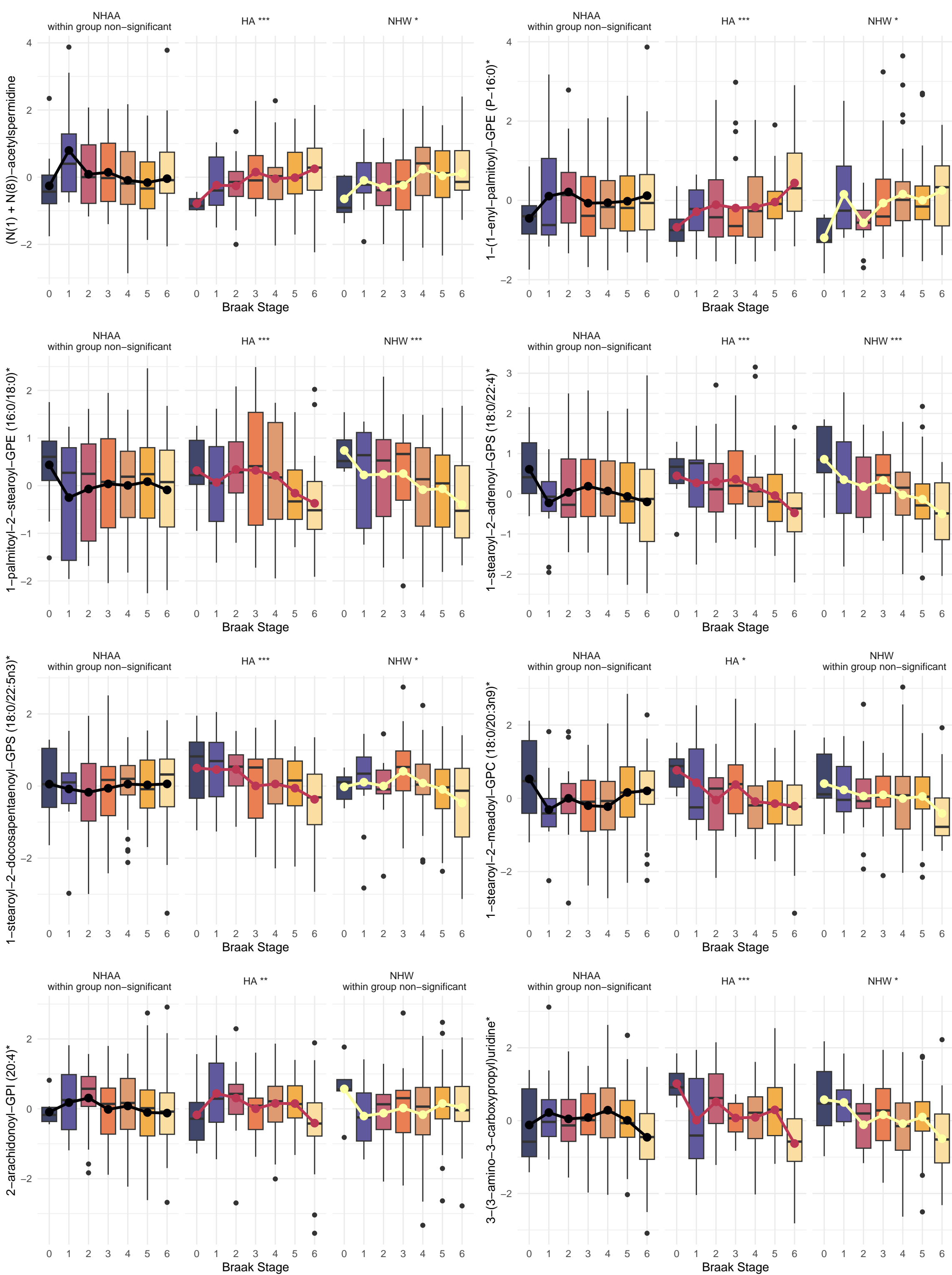

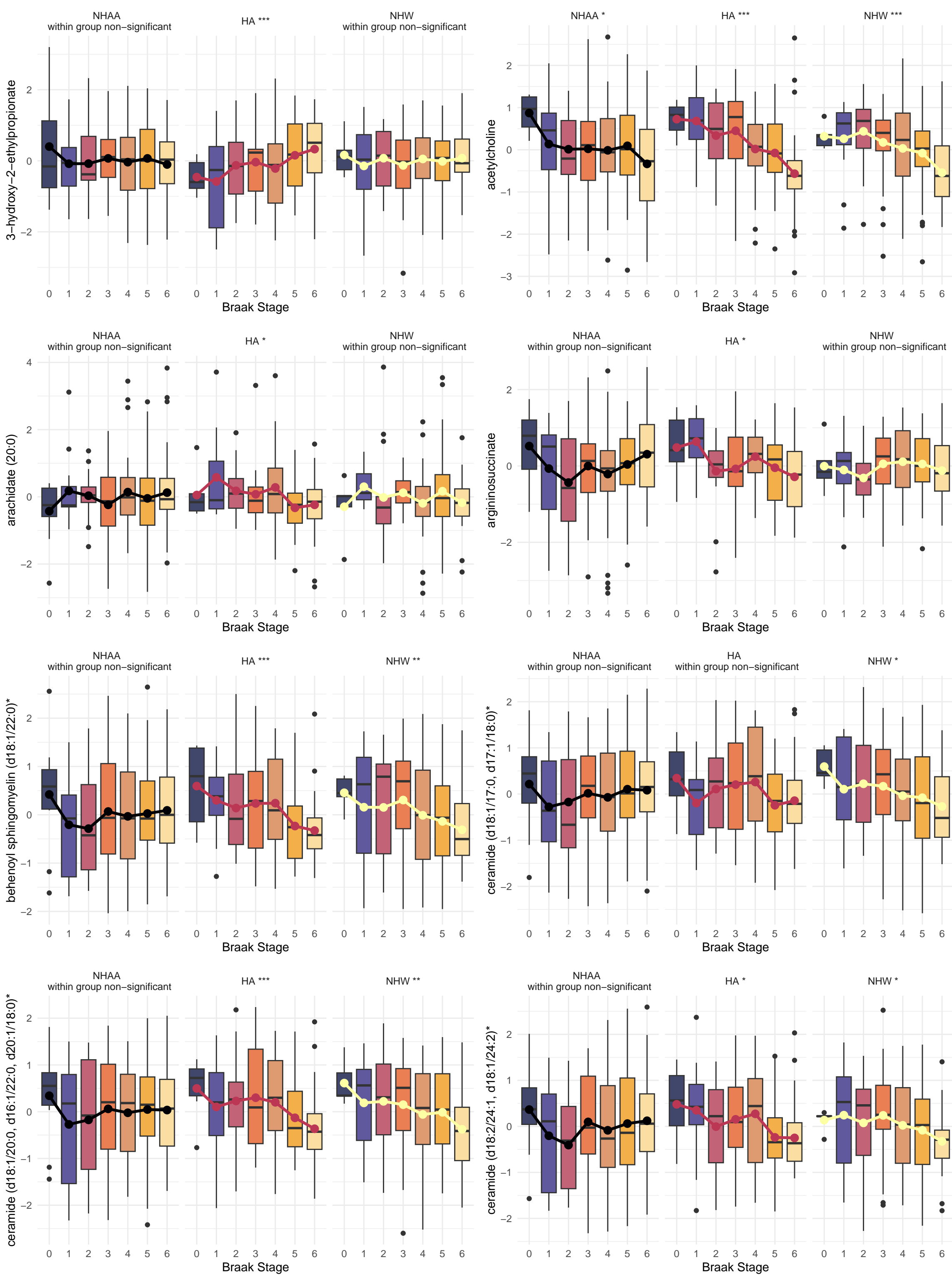

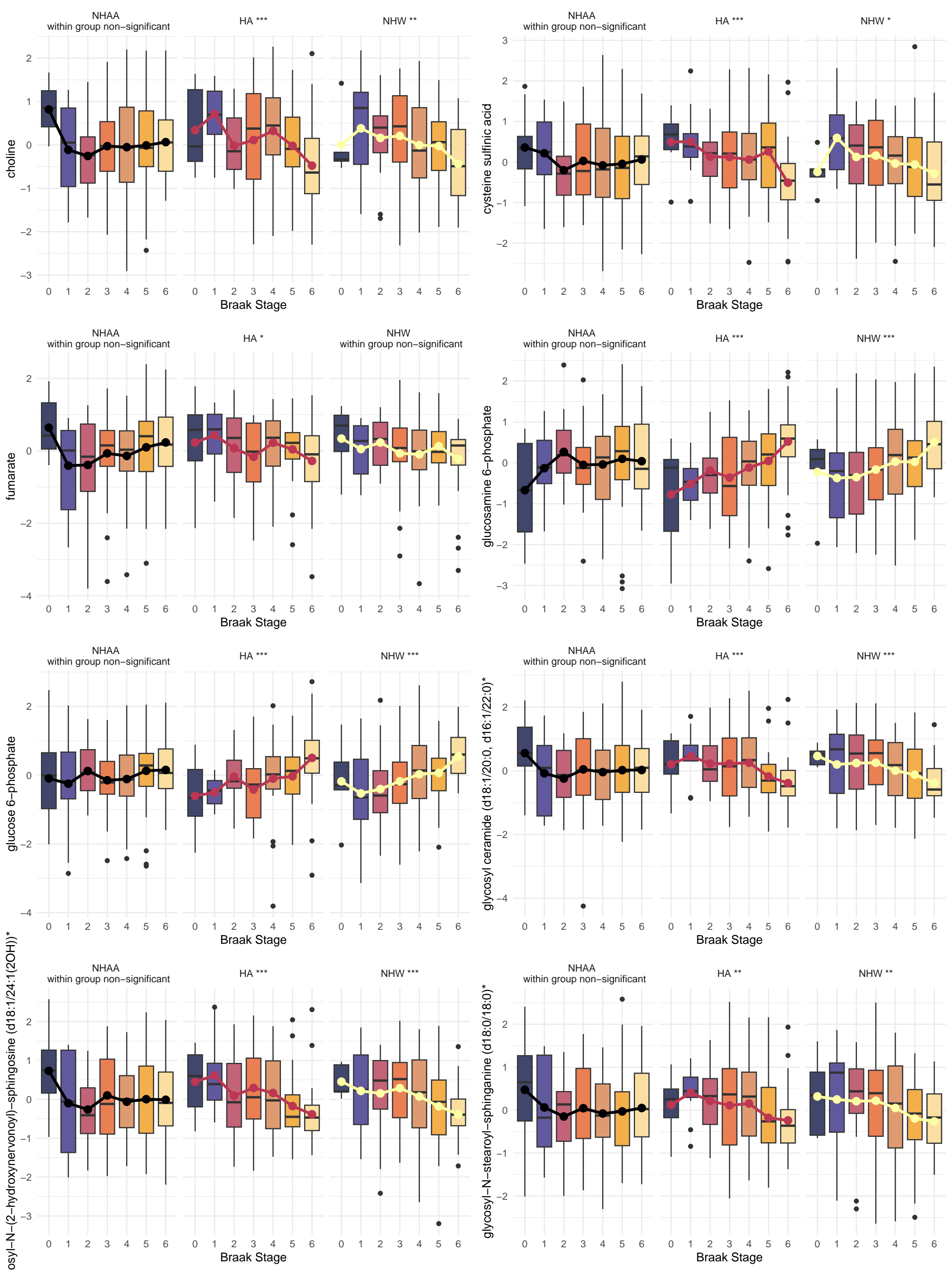

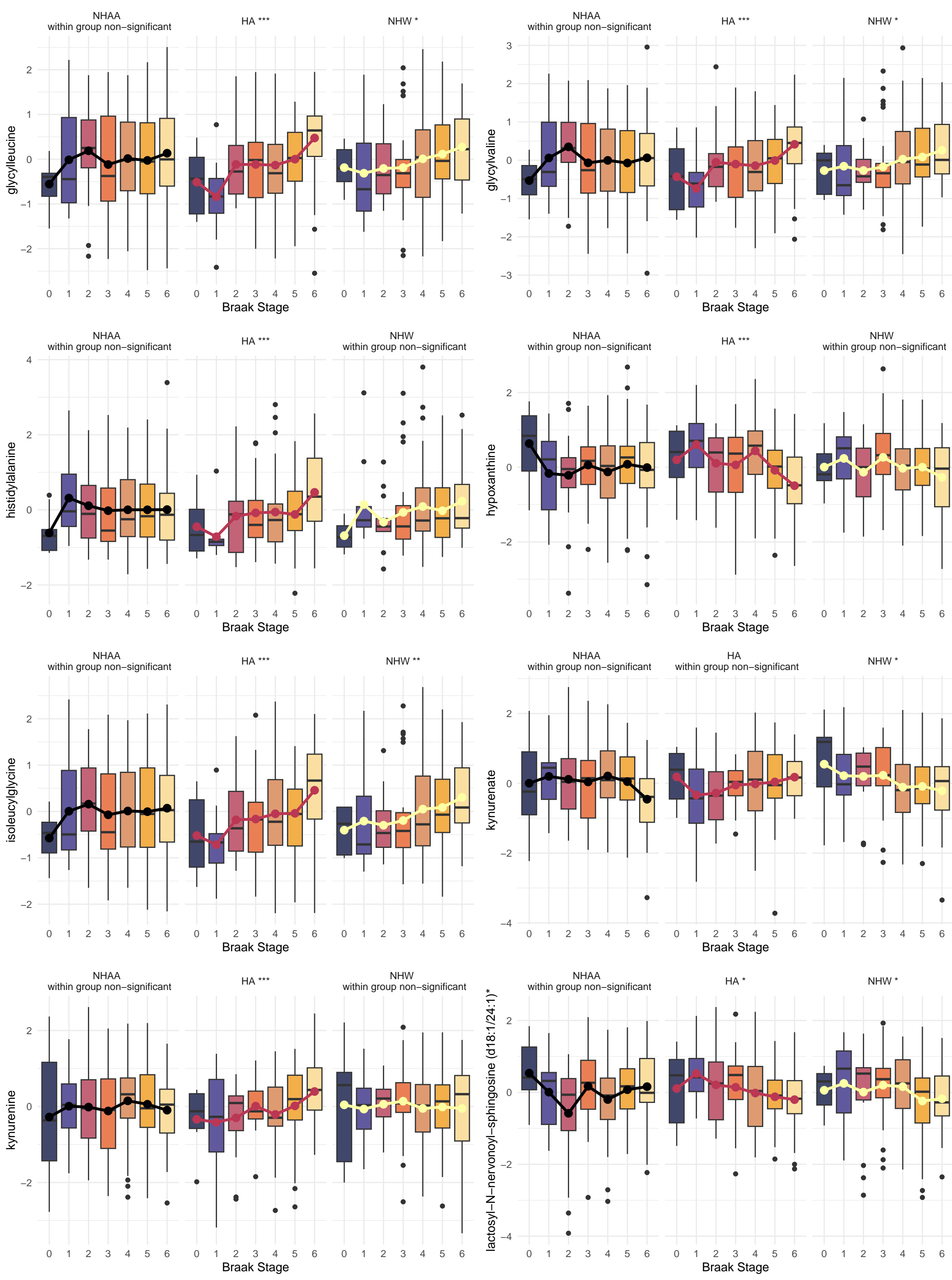

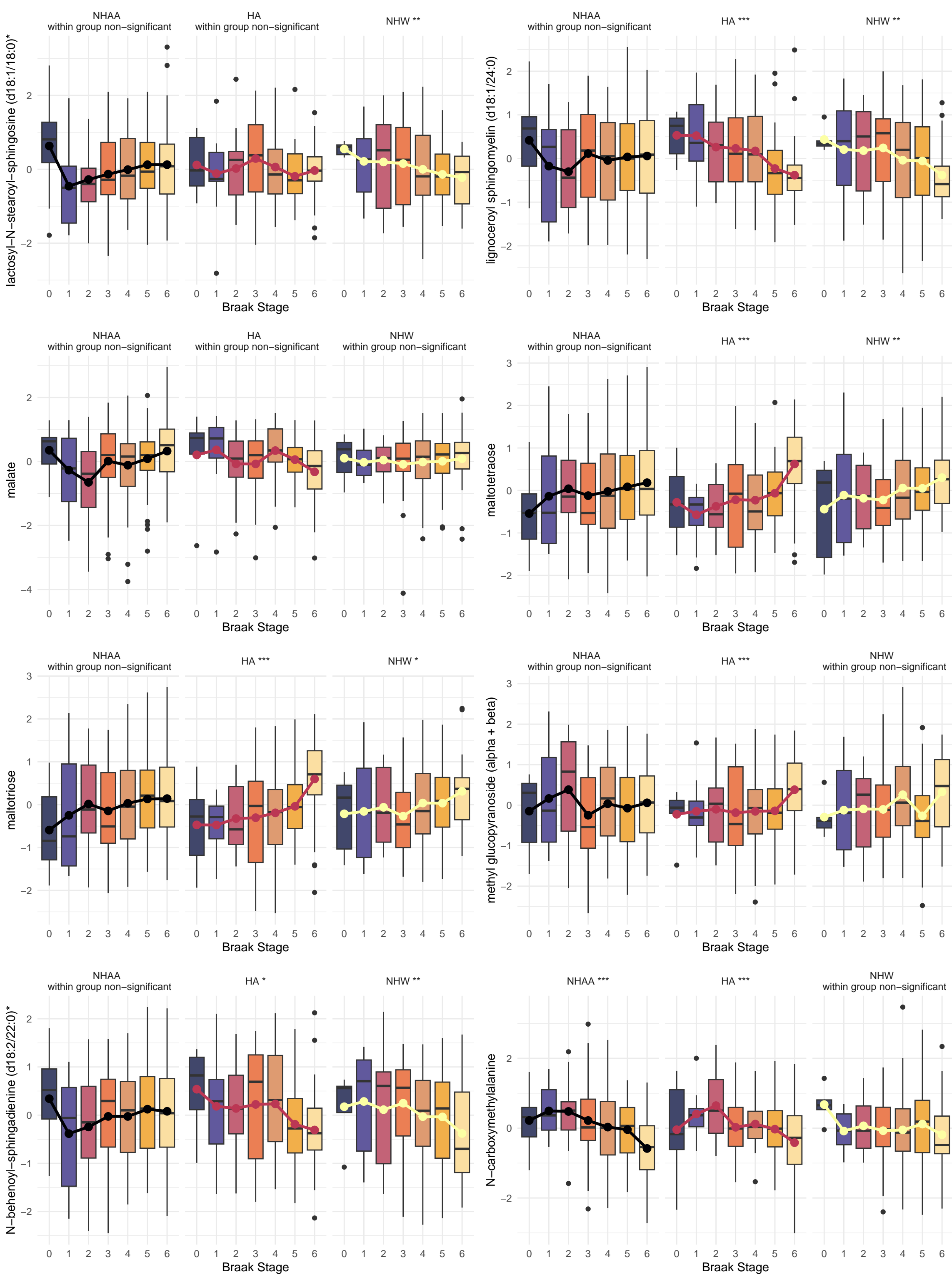

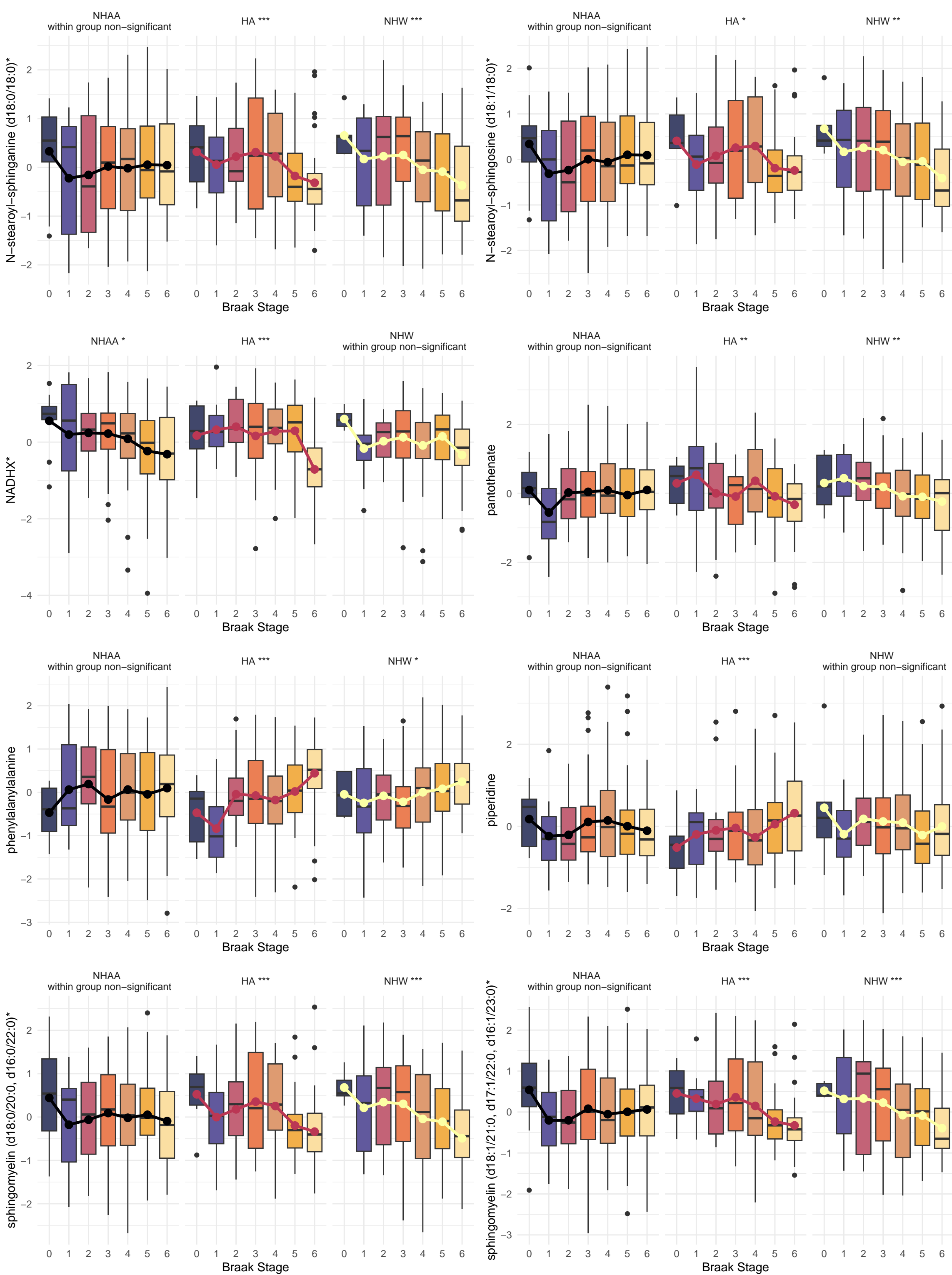

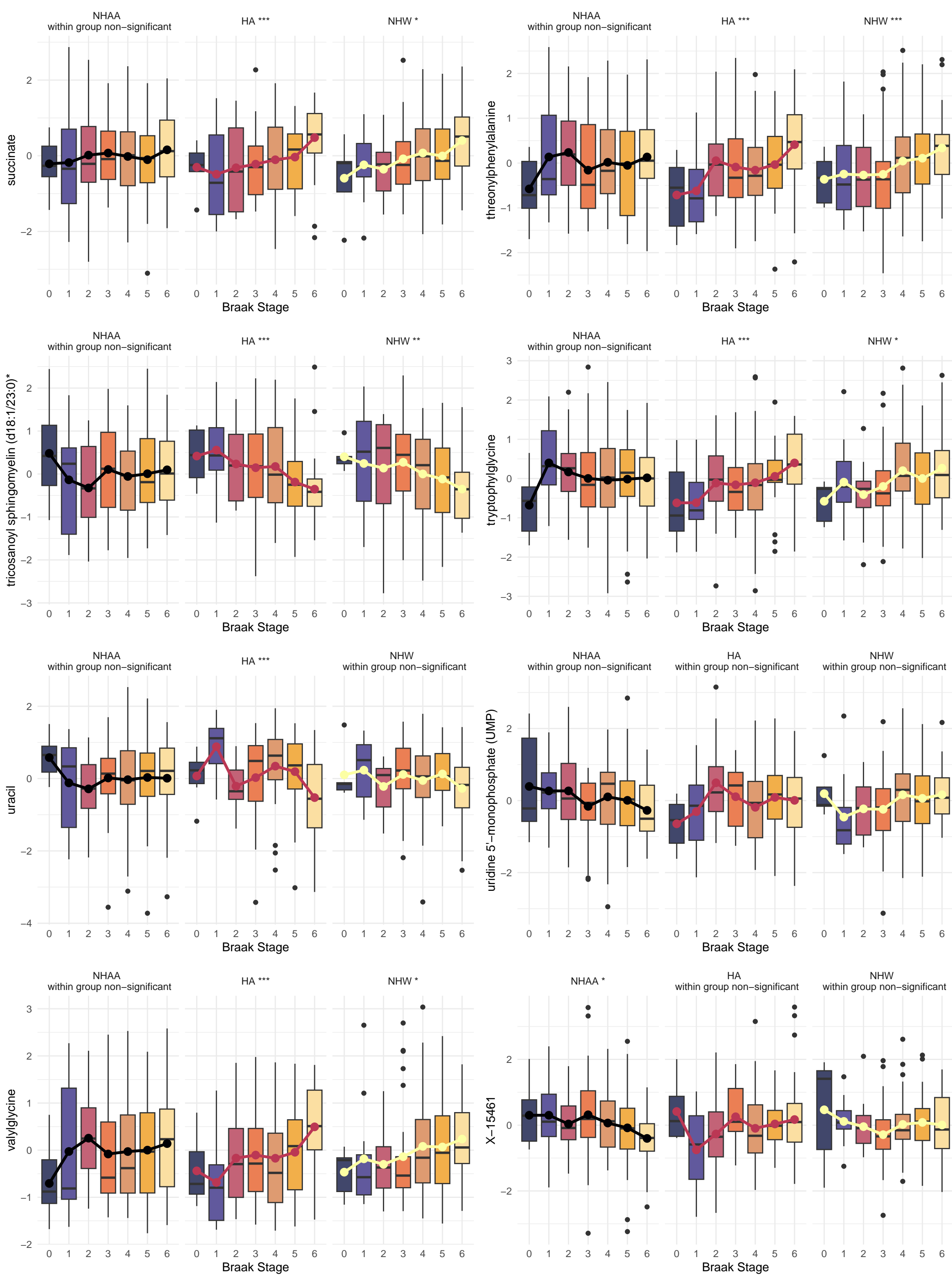

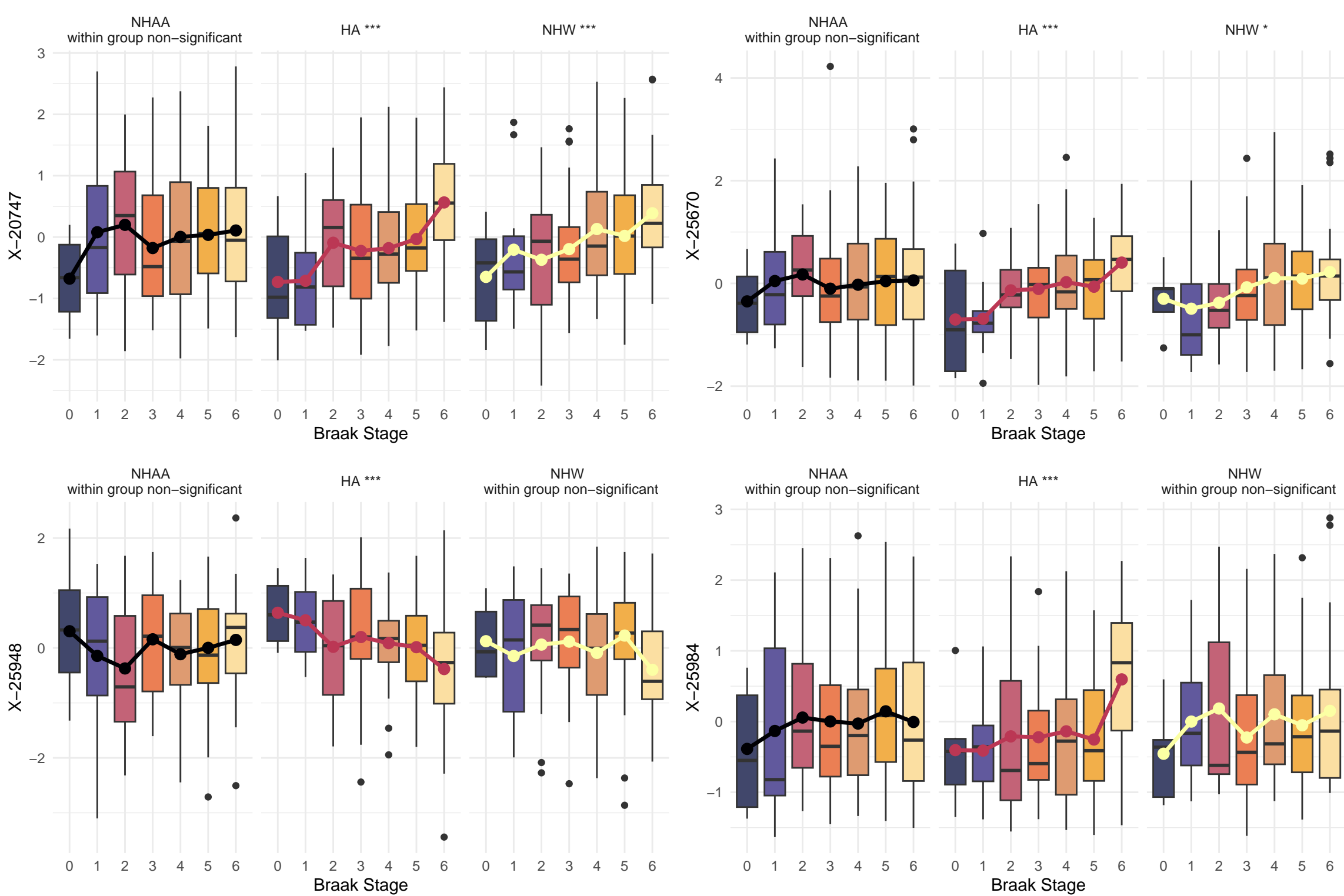

Supplement: Supplement 6 — Supplementary Figures: All metabolites with a significant interaction with Braak stage by ethnoracial group (FDR p-value < 0.1). Asterisks represent within-group significance results. ***: p < 0.005, **: p < 0.01, *: p < 0.05. [file media-6.pdf]
